# Inter-rater reliability of pressure biofeedback unit among individuals with and without chronic low back pain

**DOI:** 10.12669/pjms.38.4.4952

**Published:** 2022

**Authors:** Muhammad Khan, Hamayun Zafar, Syed Amir Gilani

**Affiliations:** 1 Dr. Muhammad Khan PT, MSPT (UK), Institute of Physical Therapy & Rehabilitation Jinnah Sindh Medical University, Karachi, Pakistan; 2 Prof. Dr. Hamayun Zafar. PT, PhD (Sweden), Department of Rehabilitation Sciences, College of Applied Medical Sciences & Medical Research Chair, King Saud University Riyadh, Saudi Arabia; 3 Prof. Dr. Syed Amir Gilani MBBS,DMRD (Pak) MPH(Pak) PhD(Ultrasound) PhD (Public Health), : University Institute of Physiotherapy, The University of Lahore, Lahore, Pakistan

**Keywords:** Pressure Biofeedback, Reliability, Low back pain, Outcome Assessment

## Abstract

**Objectives:**

To determine the inter-rater reliability of pressure biofeedback unit among individuals with and without chronic low back pain.

**Methods:**

This cross-sectional survey was conducted from February 2021 to March 2021 at the Physiotherapy Department of the Sindh Institute of Physical Medicine and Rehabilitation. Sixteen subjects which were recruited with and without chronic low back pain.(CLBP). During the test, abdominal drawing in movement was monitored by measuring a change in pressure detected in PBU. Each test was performed once by two trained assessors with period of seven days. Reliability indices of Pressure Biofeedback (PBU) measures including the Intraclass correlation coefficient [ICC] and Band Altman plot were analyzed.

**Results:**

This study found an intra-class correlation coefficient (ICC) = 0.94 [95% confidence interval (CI) (0.37, 0.99] for inter-rater reliability in healthy individuals, and an ICC of 0.97 (95% CI 0.97, 0.98) for inter-rater reliability in CLBP. The interrater agreement (Limits of Agreement–LOA=5.92, -3.9 mmHg) in CLBP and the interrater agreement (LOA=5.75, -3.25 mmHg) in healthy individuals were within the limits of agreement on 95% of occasions.

**Conclusion:**

Pressure Biofeedback Unit has showed excellent inter-rater reliability in measuring Transverse Abdominis muscle activity for individuals with and without chronic LBP.

## INTRODUCTION

Low back pain (LBP) is a symptom that is a common health problem throughout the world.^1^ Non-specific low back pain (NSLBP) is the most widespread form of LBP. NSLBP is called LBP without recognizable specific underlying pathology.^2^ Since core stability, strength and coordination provides the basis to perform smooth and coordinated upper and lower extremity movements and function, thus it is suggested that any change in the parameters providing core stability can lead to LBP.^3^ Transverses abdominis muscle strength has been shown as one of the important factors in the stabilization of the lower back. The strength of this muscle is an important factor in preventing and reducing the occurrence of low back pain.^4^ Measurement of core stability muscle strength and contraction is quite challenging, and many tools have been used to measure the strength of the abdominal muscles. These include palpation, electromyography (EMG)^5^, ultrasound imaging,^5^ Hand-Held Dynamometer^6^ and pressure biofeedback units (PBU).^7^

EMG can be used as either surface or needle EMG. However, surface EMG recording does not quantitatively measure force of muscle and deep abdominal muscles activity may not be recorded and the invasive nature of needle EMG makes it a painful procedure.^8^ Ultrasound imaging is a non-invasive tool, but this method is expensive and not easily available. Pressure Biofeedback Unit (PBU) has been designed to measure core stability muscle strength contraction. This consists of a gauge/inflation bulb which is connected to a pressure cell. This simple device measure in pressure changes during abdominal muscle contraction.^7^

A study has shown that abdominal muscle activation in standing position was higher as compared to supine position when measured with Pressure Biofeedback Unit.^9^ As far as authors knowledge is concerned only one study^10^ has evaluated the reliability of Pressure Biofeedback Unit in standing position. However, this study was carried out on asymptomatic subjects; therefore, this study has been designed to study the inter-rater reliability of PBU in standing position includes asymptomatic and subjects with low back pain.

## METHODS

This cross-sectional survey was conducted from February 2021 to March 2021 at the Sindh Institute of Physical Medicine and Rehabilitation (SIPMR). Following the approval from the Institutional Review Board (IRB-UOL-FAHS/373-VIII/2018) of the University of Lahore, non-probability purposive sampling technique was used to collect the data.

### Inclusion and Exclusion criteria:

Inclusion criteria included individuals with > 20 years of age with and without nonspecific low back pain who are willing to participate in the study and able to hold the contraction of transverse abdominus. Those who had a history of laparoscopic or spinal surgery, presence of musculoskeletal disorders or pregnancy or neurological problems such as stroke, low back pain with underlying specific cause were excluded.

After explaining the study objectives, a written informed consent Form was provided before enrolment to ask for their consent for participation in the study. The PBU is a simple device consisting of air-filled pressure bag, sphygmomanometer gauge and a catheter. The pressure bag is 16.7 × 24 cm in size and made from non-elastic material. The sphygmomanometer has a range from 20 mmHg to 100 mmHg, with the intervals of 2-mmHg on the scale. 7

Principal investigator trained the other two physiotherapists working in the SIPMR to use the PBU device adequately. Subjects were asked to stand upright with pelvis and spine in neutral position and wear a lumbar support belt firmly around the abdomen. Anterior Superior Iliac Spine (ASIS) of both sides were palpated and marked. The pressure bag was placed underneath the lumbar support one inch above the ASIS over the transversus abdominis muscle and PBU at was pumped to 70 mmHg. The participants were instructed to correctly perform abdominal drawing as much as possible and hold it for 5 seconds. For each participant 3 readings were taken and the average of these readings was recorded. Decrease in pressure values indicated the amount of Transverse abdominis muscle activation recorded by two physiotherapists on the same subjects with a duration of 7 days.

SPSS software version 23 was used to analyse the study results. Mean and S.D was presented for each quantitative variable. An intraclass correlation coefficient was calculated for the inter-rater reliability of abdominal drawing in test using the PBU. According to Fleiss’s classification, ICC values below 0.4 shows poor reliability, values in the range between 0.40 to 0.75 indicates fair to good, values above 0.75 indicates excellent reliability. Band Altman method was also used to show the mean difference of two measurements. The SEM was calculated by dividing the standard deviation of the mean differences between the two measurements by the square root of 2 and the SDC was calculated using the formula SDC= 1.96*SEM* square root of 2.

## RESULTS

In total, 16 participants were enrolled in which eight participants had CLBP patients (50% males and 50% females) and 8 healthy participants (75% Females and 25% Males). The mean and S.D of the demographic variables in CLBP were age (26.12±4.82), weight (51±10.69 kg) and height (160.33±3.94). In healthy population, mean and S.D were age (27.75±6.77), weight (54±12.18 kg) and height (160.33±3.94). The descriptive statistics of abdominal drawing-in test of the Raters in CLBP patients as well as healthy population are listed in Table-I.

**Table-I:** Mean ± SD for pressure-based unit measured in millimeters of mercury.

Chronic low back pain		
	*Mean*	*Std. Deviation*
Rater1	42.50	16.58
Rater2	41.37	17.29
Retest	41.93	16.89
Healthy population		
Rater1	48.75	6.13
Rater2	46.50	6.56
Retest	47.62	6.45

The intra-class correlation coefficient in the CLBP and Healthy population for the inter-rater reliability of PBU are shown in Table-II. In chronic low back patients, ICC value of 0.974 shows high reliability with SEM=1.76 mmHg and SDC=4.88 mmHg. The ICC value of 0.948 in Healthy population shows higher reliability with SEM=1.62 mmHg and SDC=4.5 mmHg.

**Table-II:** ICC (Inter-rater reliability) with CI.

Intraclass Correlation	95% Confidence Interval		Variance	Standard error of measurement	Smallest real difference

	Lower Bound	Upper Bound			
*Chronic low back pain*					
0.974	0.971	0.999	0.663	1.76	4.88
*Healthy population*					
0.948	0.378	0.991	1.125	1.62	4.50

**Fig.1:**
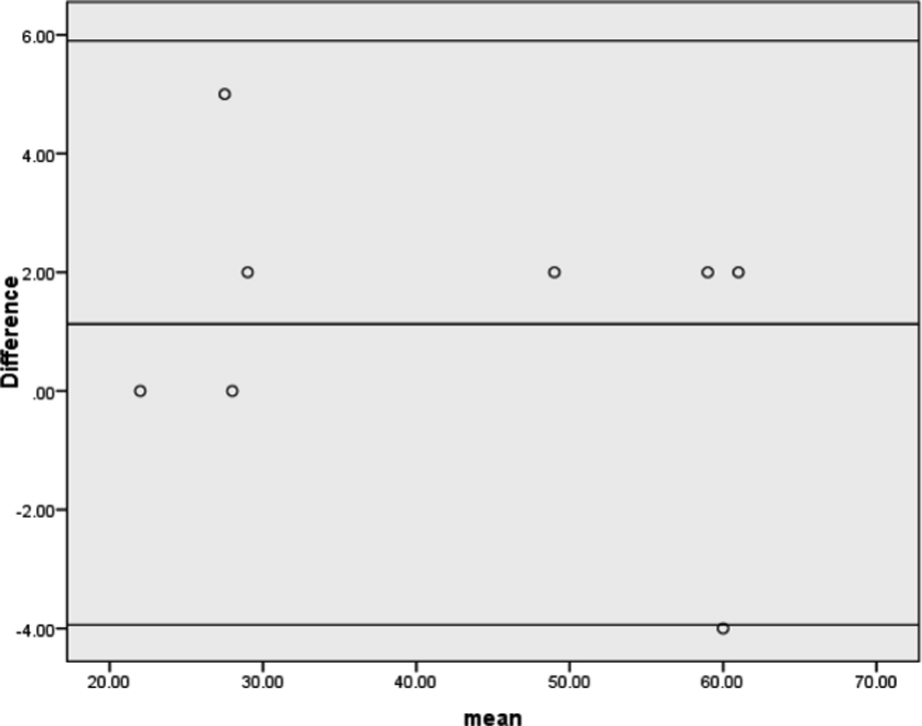
Bland-Altman limits of agreement analysis between two raters in CLBP.

The Bland-Altman chart agreement between the two raters is shown in Fig.1. Three lines are plotted on the scatter plot- one at the difference between the two raters, along with lines to plot the lower and upper limit of control limits of minus and plus 1.96*S.D. The mean difference and standard deviation of PBU in CLBP is 1.12±2.49 with a confidence interval (5.92, -3.9) and p-value p<0.05. It shows higher reliability.

**Fig.2:**
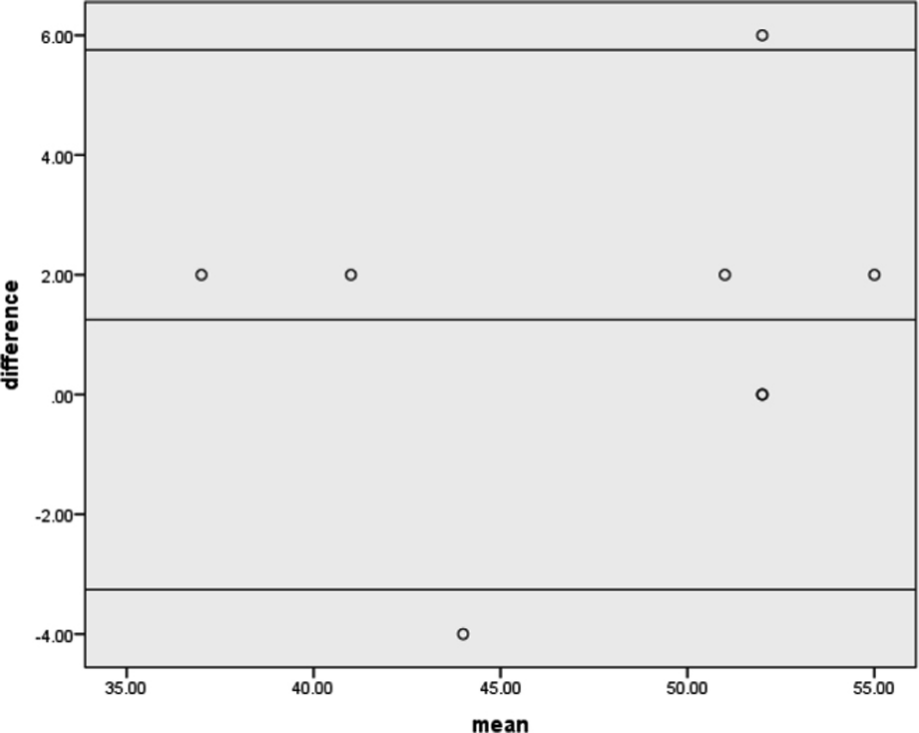
Bland-Altman limits of agreement analysis between two raters in healthy population.

The Bland–Altman method of comparison in healthy population is shown in Fig.2. Linear regression analysis detected no statistic significant drift in the difference between the tests of healthy population is 1.25±2.30 with a confidence interval (5.75, -3.25) and p-value of <0.05.

## DISCUSSION

This cross-sectional study provides evidence that PBU has excellent inter-rater reliability among individuals with and without chronic low back pain for assessing lumbopelvic stability. To the best of our knowledge, it is the first study that assessed the abdominal muscles strength in standing position among asymptomatic and subjects with low back pain. The results confirm that PBU measures can be consistently used as an outcome measure to detect the functional changes in intervention studies related to lumbopelvic stability. In current study, intertester reliability of PBU in asymptomatic individuals was 0.94, which is consistent with the findings of Dissanguan et al, who have reported reliability of 0.94.^10^ Both of these studies assessed the rectus abdominis muscle activity is standing position in young healthy participants. The present study also included participants with low back pain and found ICC= 0.97. Dissanguan et al, study also included ultrasound scan measurement and values of the PBU significantly correlated with measurements taken with ultrasound imaging. These findings are in line with Lee et al. study that used pressure feedback unit and ultrasound imaging for the training of transverse abdominis. The results have shown that 15 minutes of training significantly increased transverse abdominis thickness as compared to manual contact.^11^ In current study, intertester reliability of PBU in asymptomatic individuals was 0.94, which is inconsistent with the findings of Dilipbhai et al, who documented reliability of 0.69.^12^ The present finding is not in line with the result of Solanki et al. who reported an interrater reliability of 0.87.^13^ These findings are supported by Rathod study who reported ICC=0.89.^14^ Plausible reasons for this inconsistency might be the lower mean age, sample size and different assessment method of Transverses abdominis.

The inter-rater reliability of PBU in this study was ICC=0.97, which is not parallel with Von Garnier et al.^15^ Their study reported the interrater reliability of ICC=0.47 and the finding of the study of Figueiredo et al was ICC=0.82.^16^ Our study had a relatively small sample with the assessment carried out in standing position, which might be the reason for the variation in the ICC values across these studies.

A systematic review conducted on the clinimetric properties of PBU specifically on the evaluation of Transverse abdominus. However, difficulty in adopting the findings of the study due to the limited studies targeted the interrater reliability of PBU ranged from 0.47 to 0.82 and estimates of measurement error for clinical interpretation.^17^ In our study, SEM and SDC in chronic low back pain was 1.76 mmHg and 4.88 mmHg; SEM and SDC in healthy individuals were SEM=1.62 mmHg and SDC=4.5 mmHg. Thus, it may provide the basis to detect the real change above measurement error in upgrading the everyday clinical practice.

The time interval between tests of seven days was mentioned in two studies^16^,^18^ and one study mentioned readings were taken on the same day.^12^ To avoid the memorization of data by examiners, the ideal time duration between two reading should be at least one or two weeks.^16^,^19^ In this study, this lacking was considered while conducting the study with the period of seven days.

### Limitations of the study:

In terms of limitations, the current study was single centre and the sample size was small. Further studies need to be conducted on a larger sample size to validate the findings supported with surface EMG recording and ultrasound imaging.

## CONCLUSION

Pressure Biofeedback Unit has excellent inter-rater reliability in the measurement of Transverse Abdominis muscle activity in individuals with and without chronic nonspecific low back pain.

### Authors’ Contribution:

**MK** topic selection, designed, data collection, analysis & manuscript writing.

**HZ** topic selection, designed, manuscript review & final approval.

**SAG** editing & review of manuscript.

**MK** takes the responsibility and is accountable for all aspects of the work in ensuring that questions related to the accuracy or integrity of any part of the work are appropriately investigated and resolved.

## References

[1] Beyera GK, O'Brien J, Campbell S (2019). Health-care utilisation for low back pain:a systematic review and meta-analysis of population-based observational studies. Rheumatol Int.

[2] Maher C, Underwood M, Buchbinder R (2017). Non-specific low back pain. Lancet.

[3] Sung PS (2013). Disability and back muscle fatigability changes following two therapeutic exercise interventions in participants with recurrent low back pain. Med Sci Monit.

[4] Djordjevic O, Konstantinovic L, Miljkovic N, Bijelic G (2015). Relationship between electromyographic signal amplitude and thickness change of the trunk muscles in patients with and without low back pain. Clin J Pain.

[5] Zheng Y, Ke S, Lin C, Li X, Liu C, Wu Y (2019). Effect of Core Stability Training Monitored by Rehabilitative Ultrasound Image and Surface Electromyogram in Local Core Muscles of Healthy People. Pain Res Manag.

[6] Tanveer F, Arsalan SA, Darain H, Ahmed A (2021). Reliability of Hand-Held Dynamometer for assessing Isometric Lumbar Muscles Strength in Asymptomatic Healthy Population. Pak J Med Sci.

[7] Pienaar AW, Barnard JG (2017). Development, validity and reliability of a new pressure air biofeedback device (PAB) for measuring isometric extension strength of the lumbar spine. J Med Eng Technol.

[8] Panjabi MM (2003). Clinical spinal instability and low back pain. J Electromyography Kinesiol.

[9] Jung D, Kim K, Lee S (2014). Comparison of Muscle Activities Using a Pressure Biofeedback Unit during Abdominal Muscle Training Performed by Normal Adults in the Standing and Supine Positions. J Phys Ther Sci.

[10] Dissanguan D, Sitilertpisan P, Kiatwattanacharoen S, Joseph LH, Puangmali P, Aatit Paungmali A (2019). Reliability and Validity of the Feedback Sensor for Activating the Transversus Abdominis Muscle. Open Biomed Eng J.

[11] Lee S, Han S, Lee D (2016). Comparison of abdominal muscle thickness according to feedback method used during abdominal hollowing exercise. J Phys Ther Sci.

[12] Dilipbhai JK, Dibyendunarayan B, Ramalingam T (2016). Intrarater and Interrater Reliability of Abdominal Drawing-In Test in asymptomatic individuals. Rom J Phys Ther.

[13] Solanki VD, Soni N (2021). Interrater and Intrarater reliability of pressure biofeedback unit in measurement of transverses abdominis muscle activation in asymptomatic adults. Int J Adv Res.

[14] Rathod S (2016). Interrater and Intrarater reliability of pressure biofeedback unit in measurement of transverses abdominis activity. Indian J Physical Ther.

[15] Von Garnier K, Koveker K, Rackwitz B, Kober U, Wilke S, Ewert T (2009). Reliability of a testbmeasuring transversus abdominis muscle recruitment with a pressure biofeedback unit. Physiotherapy.

[16] Figueiredo MK, Chaves Junior IP, Figueiredo VGC, Costa LOP, Costa LCM (2005). Estudo da conabilidade intra e entre-examinadores da unidade de biofeedback pressorico na medida da contração do músculo transverso abdominal. R bras Ci e Mov.

[17] de Paula Lima PO, de Oliveira RR, Costa LO, Laurentino GE (2011). Measurement properties of the pressure biofeedback unit in the evaluation of transversus abdominis muscle activity:a systematic review. Physiotherapy.

[18] Storheim K, Bo K, Pederstad O, Jahnsen R (2002). Intra-tester reproducibility of pressure biofeedback in measurement of transversus abdominis function. Physiotherapy research international. J Res Clin Physical Ther.

[19] Terwee CB, Bot SD, de Boer MR, van der Windt DA, Knol DL, Dekker J (2007). Quality criteria were proposed for measurement properties of health status questionnaires. J Clin Epidemiol.

